# Role of Cod Liver Oil in Preventing Myocardial Infarction

**DOI:** 10.7759/cureus.16067

**Published:** 2021-06-30

**Authors:** Madho Mal, Ashok Kumar, Areeba Meraj, Arooj Devi, Alyanna Marie B Mañego, Zauraiz Anjum, Sidra Naz, Amna Jamil, Aliya Fatima, Besham Kumar

**Affiliations:** 1 Internal Medicine, Liaquat University of Medical and Health Sciences, Jamshoro, PAK; 2 Internal Medicine, Dow University of Health Sciences, Karachi, PAK; 3 Oncology, Ghulam Muhammad Mahar Medical College, Sukkur, PAK; 4 Internal Medicine, University of Santo Tomas, Muntinlupa City, PHL; 5 Internal Medicine, Fatima Jinnah Medical University, Lahore, PAK; 6 Internal Medicine, University of Health Sciences, Lahore, PAK; 7 Internal Medicine, Jinnah Postgraduate Medical Centre, Karachi, PAK

**Keywords:** myocardial infarction, prevention, cod liver oil, supplementary medicine, primary prevention

## Abstract

Introduction: Omega-3 fatty acids have for long been shown to reduce the incidence of cardiovascular (CV) diseases. Omega-3 fatty acids mainly exist in the form of eicosapentaenoic acid (EPA) and docosahexaenoic acid in fish oils. Cod liver oil is found to have a high concentration of these omega-3 fatty acids. This study aims to explore the benefits of using cod liver oil in reducing the incidence of myocardial infarction (MI) among at-risk patients.

Method: This open-label placebo-controlled two-arm interventional study was conducted in the internal medicine and cardiology unit of tertiary care hospital between January 2018 to January 2021. During this period, 870 patients at risk of CV events were enrolled in the study after obtaining informed consent. The study group received 415 mg cod liver oil daily, in addition to their current treatment, in a bottle without label and the control group received no additional treatment to their standard treatment. Patients were followed up for 12 months or till the development of MI.

Result: Patients treated with cod liver oil had comparatively fewer incidences of MI; however, the difference was not significant (p-value: 0.09). Furthermore, the difference was non-significant for both fatal and non-fatal MI. The relative risk for total MI incidence was 0.70 (0.44-1.10).

Conclusion: According to our study, adding cod liver oil to the diet does not play a major role in reducing the risk of MI. Further large-scale studies are needed to understand the role of cod liver oil in reducing the risk of CV events, including MI.

## Introduction

Myocardial infarction (MI) is defined as irreversible damage due to myocardial necrosis in the setting of myocardial ischemia. Its diagnosis is established by the presence of new-onset ischemic symptoms, electrocardiogram changes, the elevation of cardiac biomarkers, and imaging evidence of new loss of viable myocardium in the form of regional wall motion abnormality [1]. Risk factors for MI can be divided into modifiable and non-modifiable factors. Nonmodifiable risk factors include age, sex, and family history. Among the modifiable risk factors, smoking, diabetes, hypertension, and hyperlipidemia are the predominant factors [2]. The incorporation of cod liver oil into the diets of predisposed patients has shown various beneficial effects, ranging from mild reduction in hypertension to decreased synthesis of low-density lipoprotein, leading to a decrease in the risk of MI [3]. 

Omega-3 fatty acids have for long been shown to reduce the incidence of cardiovascular (CV) diseases. Omega-3 fatty acids mainly exist in the form of eicosapentaenoic acid (EPA) and docosahexaenoic acid in fish oils. Cod liver oil is found to have a high concentration of these omega-3 fatty acids [4]. It has been proposed that dietary EPA works by reducing the risk of arterial thrombosis and protects the myocardium by reducing local inflammation [5]. A study conducted on hyperlipidemic pigs demonstrated a reduction in the development of atherosclerosis in pigs that were supplemented with cod liver oil [6]. In another study, patients suspected of an acute MI were supplemented with fish oils rich in EPA. The results of the study showed that after one year, patients had lesser cardiac events and cardiac deaths as compared to the control group [7].

Sufficient data is not available demonstrating the qualitative effects of cod liver oil, in particular for the prevention of MI. This study aims to explore the benefits of using cod liver oil in reducing the incidence of MI among at-risk patients.

## Materials and methods

This open-label placebo-controlled two-arm interventional study was conducted in the internal medicine and cardiology unit of tertiary care hospital between January 2018 to January 2021. During this period, 870 patients at risk of CV events were enrolled in the study after obtaining informed consent. Ethical review board approval was taken from the institute before enrollment of the patient. Privacy and confidentiality were maintained at all costs in accordance with principles laid down in the Helsinki Declaration of Bioethics. The risk was calculated via the American Heart Association/American College of Cardiology approved risk calculator, atherosclerotic cardiovascular disease risk estimator (ASCVD risk estimator), available online at https://tools.acc.org/ascvd-risk-estimator-plus/#!/calculate/estimate/. Patients with moderate and high risk, as per the ASCVD risk estimator, were enrolled using a consecutive convenient non-probability sampling technique. Participants were randomized into two groups by 1:1 ratio using an online randomizer, research randomizer (https://www.randomizer.org/) software. The study group received 415 mg cod liver oil daily, in addition to their current treatment, in a bottle without label and the control group received no additional treatment to their current treatment. Current treatment of participants included anti-hypertensive, anti-platelets, and statins.

Age, gender, history of smoking, blood pressure, previous and family history for MI, and their current treatment regime were noted in a self-structured questionnaire. Patients were followed up for 12 months or till the development of MI, whichever came first.

Participants lost to follow-up in the study group and control group were 35 and 29, respectively. Only participants who completed the study were included in the final analysis. Statistical Package for Social Sciences (SPSS) software, version 23.0 (IBM Corp., Armonk, NY, USA) was used for data analysis. Continuous variables were analyzed via descriptive statistics and were presented as mean and standard deviation (SD). Categorical variables were presented by percentages and frequencies. Relative risk (RR) was calculated via an online calculator (MedCalc) using a 95% confidence interval. A p-value of less than 0.05 meant that there is a difference between the two groups and the null hypothesis is not valid.

## Results

The mean age of participants in the group treated with cod liver oil was 51 ± 14 while that treated without cod liver oil was 50 ± 15. No significant difference was found between the characteristics, risk factor profile, and preventive treatment in participants of both groups (Table 1).

**Table 1:** Comparison of demographics and treatment for primary prevention in both groups ACEIs: angiotensin-converting-enzyme inhibitors, ARBs: angiotensin II receptor blockers, BMI: body mass index, CCBs: calcium channel blockers, DM: diabetes mellitus, MI: myocardial infarction, NS: nonsignificant

Characteristics	Patients with cod liver oil (n= 400)	Patients without cod liver oil (n= 406)	p-value
Age in year (Mean ±SD)	51 ± 14	50 ± 15	NS
Male (%)	212 (53.0%)	221 (54.4%)	NS
Hypertension (%)	371 (92.8%)	369 (90.9%)	NS
Hypercholesterolemia (%)	291 (72.8%)	301 (74.1%)	NS
Smoking (%)	151 (37.8%)	148 (36.5%)	NS
DM (%)	251 (62.8%)	271 (66.7%)	NS
BMI greater than 25 kg/m^2 ^(%)	116 (29.0%)	121 (29.8%)	NS
Previous history of acute MI (%)	25 (6.3%)	26 (6.4%)	NS
Family history of acute MI (%)	20 (5.0%)	22 (5.4%)	NS
Treatment for Primary Prevention			
ACEIs/ARBs	381 (95.3%)	386 (95.1%)	NS
Diuretics	112 (28.0%)	109 (26.8%)	NS
CCBs	221 (55.3%)	217 (53.4%)	NS
Beta Blockers	101 (25.3%)	99 (24.4%)	NS
Statins	321 (80.3%)	319 (78.6%)	NS
Aspirin	287 (71.8%)	286 (70.4%)	NS
Clopidogrel	256 (64.0%)	260 (64.0%)	NS

Patients treated with cod liver oil had comparatively fewer incidences of MI; however, the difference was not significant (7.1% vs. 10.3%; p-value: 0.09). Furthermore, the difference was non-significant for both fatal and non-fatal MI. The RR for total MI incidence was 0.70 (0.44-1.10) (Table 2).

**Table 2:** Outcome in patients with and without treatment with cod liver oil MI: myocardial infarction

Myocardial Infarction	Patients with cod liver oil (n=400)	Patients without cod liver oil (n=406)	Relative risk Reduction (CI 95%)	p-value
Total MI	29 (7.2%)	42 (10.3)%	0.70 (0.44 to 1.10)	0.1
Non-Fatal MI	25 (6.2%)	31 (7.6%)	0.81 (0.49 to 1.36)	0.44
Fatal MI	04 (1.0%)	11 (2.7%)	0.3 (0.11 to 1.14)	0.08

## Discussion

The results of our study highlighted the role of cod liver oil in preventing MI. It demonstrated a fewer incidence of MI in patients who were given cod liver oil. However, the difference between fatal and non-fatal MI was not significant. 

Several studies correlating cod fish oil and mortality from coronary artery disease (CAD) have been conducted. A protective association was found between the usage of omega-3 polyunsaturated fatty acid (PUFA) supplement and CAD mortality in the UK population-based cohort study where dietary intake of fish was low [8]. It demonstrated that the usage of 250-500 mg/day of omega-3 PUFA, a dose which corresponds to the intake from food and supplements of 300 mg/day, resulted in a risk reduction by 25% or more [8]. Moreover, its use was associated with a 26% lower incidence of CAD mortality compared with the ones who were not using the omega-3 PUFA supplement [9]. The AGES study in Iceland, where the majority of women had approximately two to four fish portions per week in their diet, observed a lower incidence of hospitalization due to CAD [10]. All the nutrients that would be obtained when consuming fish or the other meal components consumed with it, e.g. vegetables or red meat, cannot be obtained from fish oil supplements solely, [11,12], but these supplements contain lower concentrations of contamination [13] and provide essential fatty acids which help in lowering the incidence of CAD. 

The cardiometabolic effects of omega-3 fatty acids is an active area of research. It reduces the content of arachidonic acid (AA) in membrane phospholipids in platelets, endothelial cells, and inflammatory cells with a resultant reduced production of AA-derived pro-inflammatory mediators [14]. Omega-3 fatty acids decrease the risk of thrombosis by inhibiting thromboxane A2 (TXA2) synthesis and acts as antagonists of the pro-aggregatory TXA2/prostaglandin H2 receptor in human platelets in vitro [15]. Moreover, it also decreases very low-density lipoprotein assembly and secretion, resulting in reduced triacylglycerol production, through a decreased activity of sterol receptor element-binding protein-1c. On the other hand, it exerts antiarrhythmic effects by inhibiting voltage-gated sodium channels, prolonging the relative refractory period, and increased voltage that is required for membrane depolarization [16]. All of these properties of omega-3 fatty acids have attracted much attention towards its consumption in reducing cardiac events [17].

To the best of our knowledge, this is the first study in a local setting that studies the role of COD liver oil in preventing MI. However, since the study was conducted only in one institute, care should be taken while inferring its result to a larger population. Another limitation was that our study only followed patients for the development of MI and did not measure the impact of COD liver oil on risk factors associated with MI due to lack of resources.

## Conclusions

In conclusion, according to our study, adding cod liver oil to the diet does not play a major role in reducing the risk of MI. However, based on literature, it may have a role in improving various risk factors associated with MI. Further large-scale studies are needed to understand the role of cod liver oil in reducing the risk of CV events, including MI. These studies should aim to emphasize the need for its intake to inhibit atherosclerotic events and to prevent coronary disease in highly susceptible people.
